# Impact of sintering temperature and compression load on the crystallinity and structural ordering of polytetrafluoroethylene

**DOI:** 10.1039/d5ra03395k

**Published:** 2025-09-10

**Authors:** Elham Katoueizaheh, Hossein Rajabinejad, Aran Rafferty, Michael A. Morris

**Affiliations:** a School of Chemistry, CRANN and AMBER Research Centres, Trinity College Dublin Dublin 2 Ireland rajabinh@tcd.ie

## Abstract

Polytetrafluoroethylene (PTFE) parts are normally consolidated under multi-tonne presses and sintered, but the separate roles of load and temperature on crystal development remain under-explored. Here, we molded PTFE powder under compressive loads (1.2, 1.8 and 2.4 kN) followed by sintering (320, 330 and 340 °C). The starting powder and molded films were characterised using X-Ray Diffraction (XRD), Fourier-Transform Infrared Spectroscopy (FTIR), Small-Angle X-Ray Scattering (SAXS), Dynamic Image Analysis (DIA) and Scanning Electron Microscopy (SEM). Raising the sintering temperature from 320 °C to 340 °C (at 1.8 kN) increased XRD-derived crystallinity from 63.5% to 71.8% and sharpened five hexagonal-phase reflections ((100)–(210)), while the *a* and *c* lattice parameters contracted by ∼0.3% and ∼1.1%, respectively, indicating tighter chain packing. SAXS revealed a concomitant 7% reduction in long-period spacing, and FTIR showed intensified CF_2_ and C–C bands, signifying enhanced chain alignment. In contrast, elevating the load from 1.2 kN to 2.4 kN at 320 °C trimmed bulk porosity from 33% to 25% without a statistically significant crystallinity change (63.0% → 64.8%). SEM cross-sections established that additional load mainly closes residual inter-particle voids rather than promoting crystal growth. Taken together, the data demonstrates that temperature is the primary driver of phase-IV hexagonal ordering, whereas load chiefly controls densification and has only a minor effect on ordering. This work, therefore, establishes a practical, low-pressure processing window 330 to 340 °C under ∼1.8 kN, which yields highly-ordered (≈72% crystalline), low-porosity (≈25%) PTFE films, providing a cost-effective route for manufacturing PTFE films and components.

## Abstract 2

This work has established a practical, low-pressure processing window which yields highly-ordered, low-porosity PTFE, providing a cost-effective route for manufacturing PTFE films and components.

## Graphical abstract

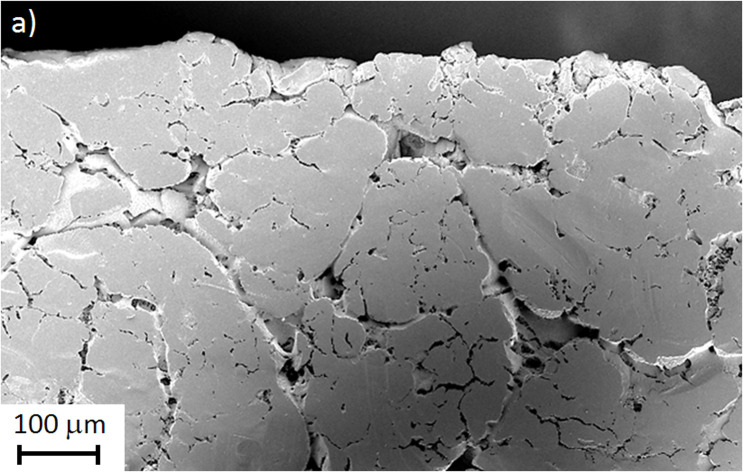

## 1 Introduction

Polytetrafluoroethylene is a semi-crystalline fluoropolymer characterized by outstanding chemical inertness, excellent thermal stability, low friction coefficient and very good dielectric characteristics.^1,2^ These properties have established PTFE as an essential material in numerous high-performance applications, including aerospace, chemical processing, electronics and biomedical engineering.^1^ The distinctive combination of robust carbon-fluorine bonds with a helical molecular configuration enhances PTFE's exceptional resistance to chemical attack, thermal deterioration and electrical failure.^3^

Despite its advantageous properties, PTFE presents significant processing challenges due to its high melting point (approximately 327 °C) and its inability to be processed through conventional melt-processing techniques.^4–6^ In contrast to traditional thermoplastics, PTFE lacks a regular melting phase and instead transitions into a gel-like condition when heated, thus complicating manufacturing with common polymer processing methods like extrusion or injection molding.^7,8^ Compression molding and sintering has become established as the primary technique for forming PTFE products, facilitating the creation of components with intricate geometries and customized characteristics.^9,10^

Compression molding involves compacting a powder to form a preform, which is subsequently sintered at elevated temperatures to promote particle coalescence and enhance mechanical integrity.^9,10^ The parameters influencing this process, particularly compression load and sintering temperature, are essential in defining the final microstructure and characteristics of the product.^10,11^ Fluctuations in these parameters can cause substantial alterations in crystallinity, crystalline phase transitions and morphological attributes, thereby affecting the material's mechanical properties, thermal stability and dielectric performance.^10,11^ It is against this backdrop that the present study explores how sintering temperature and sub-3 kN compression load separately influence crystallisation and densification in compression-molded PTFE films.

PTFE demonstrates intricate phase transition characteristics owing to its various crystalline structures, comprising phase II (triclinic), phase IV (hexagonal) and phase I (hexagonal with enhanced chain mobility). These phases can interconvert in a limited temperature range of 19–30 °C at standard pressure.^12,13^ These transitions indicate alterations in both short-range and long-range ordering of the polymer chains, influencing mechanical parameters such as tensile strength, elongation at break and fatigue resistance.^14^ The transition from phase II to phase IV entails an expansion of the helical shape and enhanced chain mobility, which may be affected by mechanical deformation and thermal history.^13^ Analytical techniques are used to investigate these effects; XRD to elucidate crystalline structure and phase composition,^15,16^ FTIR to identify molecular interactions and conformational alterations within polymer chains,^17^ SAXS to reveal morphological characteristics, including lamellar thickness and degree of orientation,^18^ Differential Scanning Calorimetry (DSC) to evaluate thermal transitions, such as melting and crystallization behaviours^19^ and, finally, SEM and DIA can aid understanding of particle size, particle shape, the propensity of particles to pack effectively, as well as the morphology of molded films.

Using a combination of the above-mentioned techniques, we examine the structural and morphological evolution of molded and sintered PTFE. Understanding the interplay between processing conditions, phase transitions and microstructure evolution is crucial, especially for components subjected to cyclic loading or extreme environmental conditions.^15,20^ As mentioned above, PTFE product durability can be significantly affected by phase transitions.^1,21^ In aerospace applications, PTFE seals and gaskets must preserve integrity at varying temperatures and pressures, with microstructural stability being critical.^22,23^ In the biomedical space, PTFE is used for mechanically reliable and biocompatible implants and prostheses.^24,25^ Research has demonstrated that the mechanical characteristics of PTFE are significantly influenced by variables like temperature, crystallinity, applied strain and stretching rate.^16,17^ The majority of existing research is focussed on the stretching characteristics of PTFE,^18,19^ with significantly less attention paid to the influence of compression molding parameters on its phase transition behaviour and crystalline structure.

Here, we decouple temperature-driven crystal growth from load-driven densification in PTFE compression molding for the first time. By confining the load to ≤2.4 kN we show that high crystallinity (≈72%) and low residual porosity (≈25%) can be achieved without the multi-tonne presses often cited in the literature. The following sections detail how our low-pressure processing window was established and validated.

## 2 Experimental

### 2.1 Materials

Polytetrafluoroethylene powder (TF2021Z) with a nominal average particle size of 500 μm and a bulk density of 475 kg m^−3^ was sourced from 3M™Dyneon. The material was used as-received.

### 2.2 Sample preparation

PTFE films were prepared by compression molding using a hydraulic press (Carver Laboratory Press Model 4386, Carver Inc.). Each sample, as detailed in Table 1, was made from 5 grams of powder, pressed in a 50 mm × 50 mm × 1 mm stainless-steel mold.

**Table 1:** The samples studied in this work

Sample	Compression load (kN)	Sintering temperature (°C)
Film S1	1.8	340
Film S2	1.8	330
Film S3	1.8	320
Film S4	1.2	320
Film S5	2.4	320
Film S6	2.4	—
Powder P7	—	—
Powder P8	—	340

Compression molding comprised the following steps:

The filled mold was placed in a hydraulic press under a load of 0.5 kN for 5 minutes to pre-compact the powder.

The platens' temperature was increased at a rate of 5 °C min^−1^ to temperatures of 320, 330, and 340 °C, respectively. The load was set to the predetermined value (1.2, 1.8, or 2.4 kN) and sustained for a duration of 60 minutes at the target sintering temperature.

Following sintering, controlled cooling took place at 5 °C min^−1^ at a fixed pressure, necessary to prevent warping and negative effects on crystallinity.

Where sintering was not conducted, the procedure entailed cold compaction alone. Powder was also analysed, as-received, and following sintering at 340 °C without compression (Table 1, samples 6–8).

### 2.3 Material characterisation

#### 2.3.1 X-ray diffraction (XRD)

Powder XRD analysis was performed to investigate crystalline structure and degree of crystallinity using a Bruker D8 Discover X-ray diffractometer equipped with a Cu Kα radiation source (*λ* = 1.5406 Å), operated at 45 kV and 40 mA. Samples were scanned over a 2*θ* range of 5° to 60° at a step size of 0.02° and a counting time of 1 second per step. The crystalline phases were identified by comparing the diffraction patterns with the International Centre for Diffraction Data (ICDD) PDF-2 database.

The degree of crystallinity, *X*_c_, was determined using the Hermans–Weidinger method:^26^1
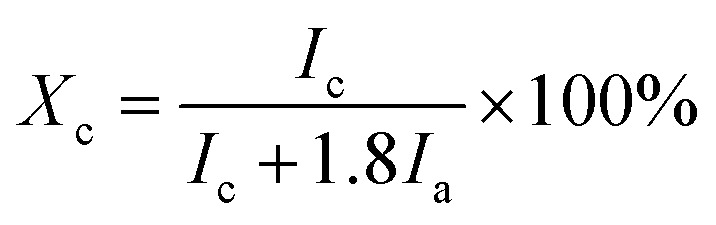
and the Scherrer equation used to estimate the crystallite size (*D*):2
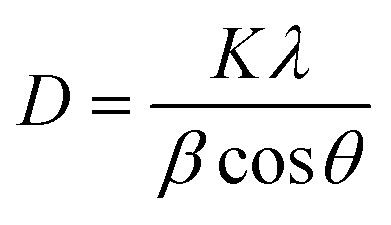
where *K* is the shape factor (typically taken as 0.89 for polymers), *λ* is the X-ray wavelength (1.5406 Å for Cu Kα radiation), *β* is the Full Width at Half Maximum (FWHM) of the diffraction peak, and *θ* is the Bragg angle.

#### 2.3.2 Fourier-transform infrared spectroscopy (FTIR)

A PerkinElmer Spectrum 100 FTIR/ATR spectrometer equipped with a built-in attenuated total reflectance (ATR) accessory featuring a diamond crystal was used over a wavenumber range 4000 to 400 cm^−1^ at a resolution of 4 cm^−1^, with background spectra recorded, as standard.

#### 2.3.3 Small-angle X-ray scattering (SAXS)

A Bruker SAXS point 2.0 system equipped with a Cu Kα radiation source was used on samples prepared as thin films. Scattering data was collected over a *q*-range 0.01 to 0.5 Å^−1^, under vacuum. Data analysis used DIFFRAC.EVA software, employing models appropriate for semi-crystalline polymers.

#### 2.3.4 Density measurements

Bulk density of films was measured according to the Archimedes method (ASTM D792-13), using an analytical balance and density determination kit (Mettler Toledo MS204S, Mettler-Toledo LLC, USA). Density was calculated using the equation:3
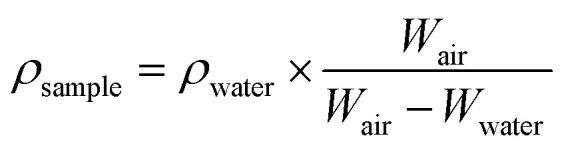
where *ρ* is density and *W* is weight.

True density of films was measured using an Ultrapyc 5000 Micro gas pycnometer (Anton Paar), with nitrogen gas at a fixed temperature of 20 °C. Porosity was calculated from the bulk and true density values, according to the equation:4
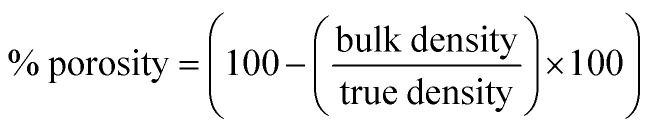


All experiments were performed in triplicate, with results expressed as mean ± standard deviation (SD). OriginPro 2021 was used for statistical analysis, curve-fitting, and plotting.

Adobe Illustrator v27.2 was used to generate conceptual 3D schematics of the formed morphology, and the various stages of compression molding and sintering, using measured particle diameters and mold dimensions as scale references.

#### 2.3.5 Scanning electron microscopy (SEM)

Scanning electron microscopy of PTFE powder was performed using a Zeiss ULTRA-plus high resolution field emission microscope capable of 1 nm resolution at 15 kV. Micrographs of molded films were acquired at 5 kV and 30 pA with a 5 nm Au coating to minimise beam-induced artefacts.

#### 2.3.6 Particle size and shape analysis

A Dynamic Image Analyser (DIA-500, Anton Paar) was used to measure particle size distribution and shape parameters. The material was analysed using a dry-jet feed system which includes the option of using compressed air (up to 4600 mbar) to aid dispersion and deagglomeration. Here, the compressed air function was utilised (15 s dry-jet burst at pressures of 16 mbar and 4000 mbar) to investigate the effect of air pressure on particle size, shape and degree of fragmentation, after which the particles were collected for SEM analysis.

**Fig. 1:**
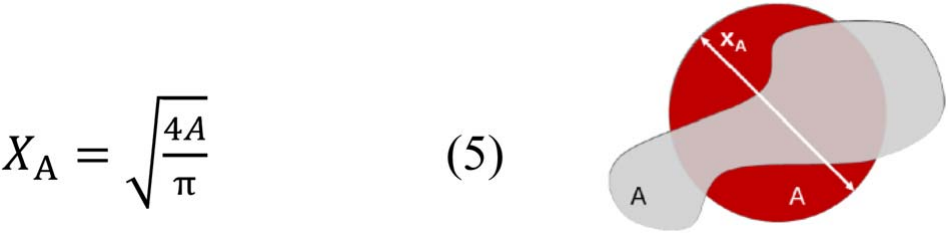
Equation for and schematic representation of projected area equivalent diameter.

Particle size distributions were expressed as *X*_*A*_, on a volume-basis, where *X*_*A*_ is defined as the diameter of a sphere with the same projected area (*A*) as the particle's projection (Fig. 1).

**Fig. 2:**
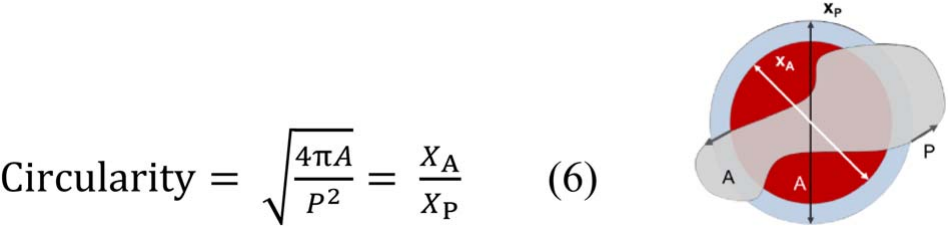
Equation for and schematic representation of circularity.

Particle shape was expressed using the circularity parameter. Circularity is defined as the degree to which the particle (or its projection area) is similar to a circle, considering the smoothness of the perimeter (*P*). It ranges from 0 to 1 (sphere) (Fig. 2).

## 3 Results and discussion

This section details how sintering temperature and ≤2.4 kN compression load independently control crystallisation and densification in PTFE, providing, for the first time, a low-pressure processing window that achieves >70% crystallinity.

### 3.1 X-ray diffraction (XRD) analysis

**Fig. 3:**
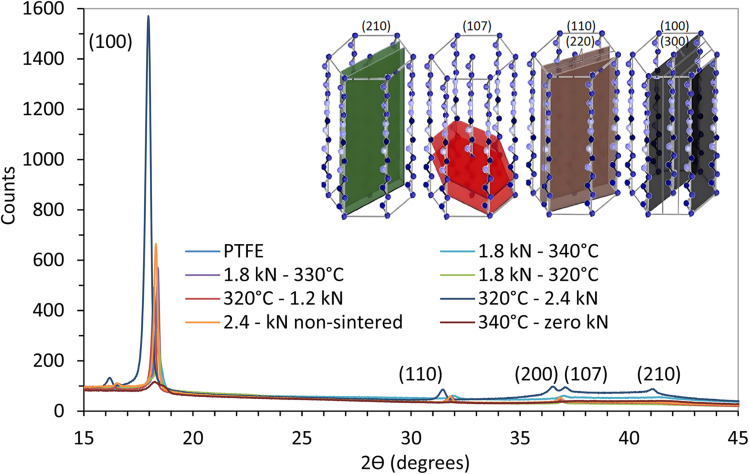
XRD diffractograms of the prepared samples.

XRD diffraction data obtained for the various samples are presented in Fig. 3.

Characteristic peaks consistent with PTFE's typical crystalline structure are observed at 2*θ* values of 18.01, 31.54, 36.58, 36.99 and 41.19°, corresponding to the (100), (110), (200), (107) and (210) planes, respectively. These diffraction peaks confirm that the samples predominantly exhibit the phase IV hexagonal arrangement.^12,27–29^

**Fig. 4:**
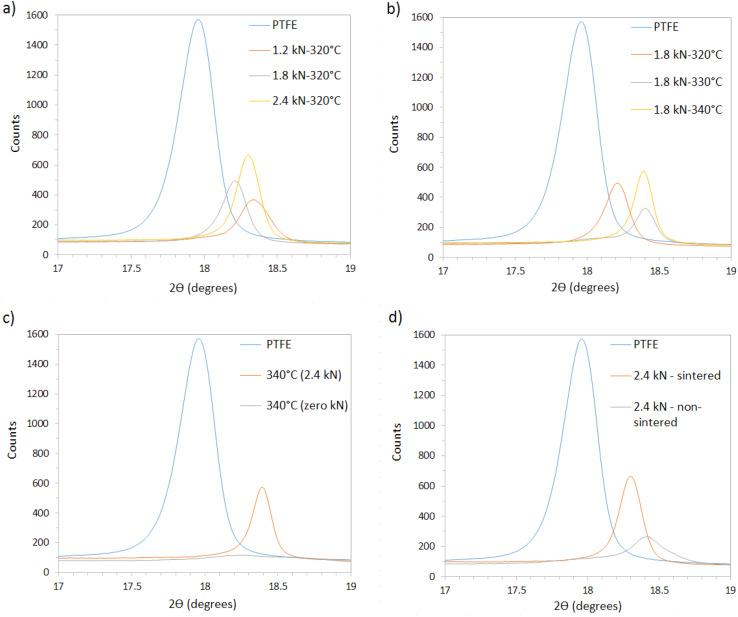
XRD spectra of samples: (a) pressed at different loads (1.2, 1.8, 2.4 kN) and sintered at 320 °C, (b) sintered at different temperatures (320, 330, 340 °C) with a constant load of 1.8 kN, (c) sintered at 340 °C, with and without compression, and (d) compressed at 2.4 kN, with and without sintering.

Fig. 4(a)–(d) compare samples subjected to different loads and sintering conditions.

Fig. 4(a) shows that, at a fixed sintering temperature of 320 °C, raising the compression load from 1.2 kN to 2.4 kN only marginally narrows the (100) reflection, confirming that pressure chiefly affects densification rather than crystal growth. Conversely, Fig. 4(b) demonstrates a pronounced sharpening and intensity gain of that same (100) peak when the sintering temperature is increased from 320 °C to 340 °C at a constant 1.8 kN, underscoring temperature as the dominant driver of crystallinity. Fig. 4(c) compares XRD patterns for samples pressed at 2.4 kN with and without sintering. The non-sintered sample displayed significantly lower crystallinity (46.0%), as indicated by the lower intensity of the crystalline peaks, and is designated as ‘amorphous’ (Table 2), emphasizing the role of sintering in forming a well-defined crystalline structure.

**Table 2:** Summary of XRD data for the various samples

Sample	Load (kN)	Sintering temp. (°C)	Crystallinity (%) ± 1% SD	*a* (Å) ± 0.003	*c* (Å) ± 0.003	Unit-cell *V* (Å^3^) ± 0.3	Phase
Film S1	1.8	340	71.8	5.655	19.508	540.3	Hexagonal
Film S2	1.8	330	67.1	5.665	19.559	543.6	Hexagonal
Film S3	1.8	320	63.5	5.672	19.729	548.2	Hexagonal
Film S4	1.2	320	63.0	5.690	19.773	553.0	Hexagonal
Film S5	2.4	320	64.8	5.622	19.506	542.3	Hexagonal
Film S6	2.4	—	46.0	N/A	N/A	N/A	Amorphous
Powder P7	—	—	61.2	5.660	19.560	543.6	Hexagonal
Powder P8	—	340	33.3	N/A	N/A	N/A	Amorphous

Higher sintering temperatures gave narrower diffraction peaks, suggesting reduced internal stresses and better-ordered crystallites. Lower sintering temperatures gave broader peaks, indicating the presence of microstructural defects and smaller crystallites.^26^ Enhanced chain mobility at higher temperatures allows for the relaxation of internal stresses and the formation of larger, more ordered crystallites.^29^

The degree of crystallinity (*X*_c_) of the samples were calculated, as summarized in Table 2. Mean ± SD values are based on three independent XRD scans; the ±1% SD implies that crystallinity differences <2% are not statistically significant.

The results show that increasing the sintering temperature enhances the degree of crystallinity, with the film sintered at 340 °C at 1.8 kN exhibiting the highest crystallinity of 71.8%. This can be attributed to additional thermal energy enabling the polymer chains to rearrange and form more ordered crystalline regions.

The effect of compression load on crystallinity was next examined. At 320 °C the crystallinity rises only from 63.0% (1.2 kN) to 64.8% (2.4 kN), a difference that lies within the ±1% experimental uncertainty of our triplicate measurements. Consequently, no statistically significant increase in crystallinity can be claimed. Overall, the effect of load on crystallinity was less pronounced compared to the effect of temperature. The data therefore, supports the view that loads below ∼3 kN mainly drive densification, while temperature controls the crystal-growth kinetics.

At 340 °C the refined hexagonal lattice parameters are *a* = 5.655 Å and *c* = 19.508 Å; at 330 °C and 320 °C they increase marginally to 5.665 Å/19.559 Å and 5.672 Å/19.729 Å, respectively. This systematic expansion of both *a* and *c* as the sintering temperature falls (≈0.3% in *a* and 1.1% in *c*) suggests slightly looser crystal packing and a small increase in free volume, consistent with the reduction in overall crystallinity observed at the lower temperatures. Taken together, the XRD data confirms that temperature is the dominant lever for crystal growth within the ≤2.4 kN range.

The intensity of the peaks varies, consistent with changes in crystallinity. This is also reflected in the peak widths (FWHM): samples with higher crystallinity exhibit narrower peaks, indicating larger crystallite sizes as determined by the Scherrer equation. For instance, samples sintered at 340 °C had an estimated crystallite size of ∼45 nm, whereas the non-sintered sample showed smaller crystallites ∼30 nm. Powder that was not compressed or sintered showed a crystallinity of 61.2%, highlighting the role of processing in enhancing crystallinity.

**Fig. 5:**
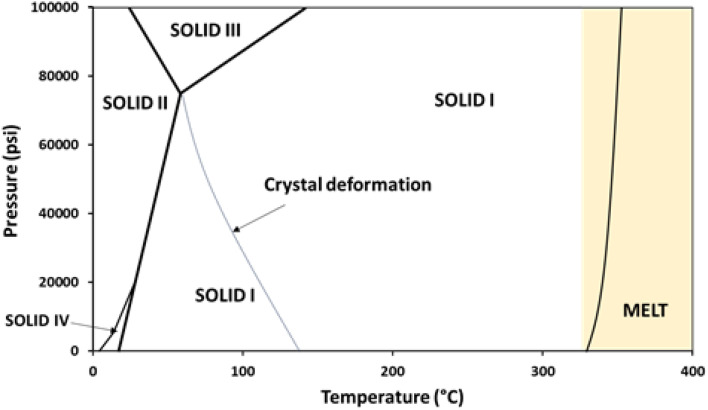
Pressure-temperature phase diagram of PTFE showing the different crystalline phases and melt phase.^23^

The PTFE phase diagram (Fig. 5) illustrates the temperature and pressure regimes under which different crystalline phases are stable. The sintering temperatures used in this study (320, 330, 340 °C) fall within the Solid I region. For lower temperatures, <140 °C, loads applied during molding could cause a shift into other solid phase regions.

No distinct phase transitions occurred for our samples; rather, the hexagonal phase was refined and became more ordered as temperature and load increased. Similar observations were reported by Brown *et al.*,^12^ who found that sintering in the Solid I region led primarily to increased ordering of the hexagonal phase, rather than a transition to a new phase. Our findings of increased crystallinity at higher temperatures are also consistent with previous studies. Brown *et al.*^12,30^ reported higher sintering temperatures enhancing chain mobility and leading to increased crystallinity. Conversely, Yuan *et al.*^31^ observed that sintering (>350 °C) can lead to thermal degradation and reduced crystallinity. In terms of compression load, Brown *et al.*^30^ reported significantly increasing crystallinity for specimens compacted under hot-press loads of 50–150 kN (on 12 mm-diameter billets), far higher, and on a larger scale, than the 1.2–2.4 kN range employed here. The two-orders-of-magnitude difference in applied load, together with the use of higher-crystallinity PTFE 7C powder, likely accounts for the larger crystallinity gains reported by Brown *et al.*^12^

### 3.2 Size and shape of PTFE powder

**Fig. 6:**
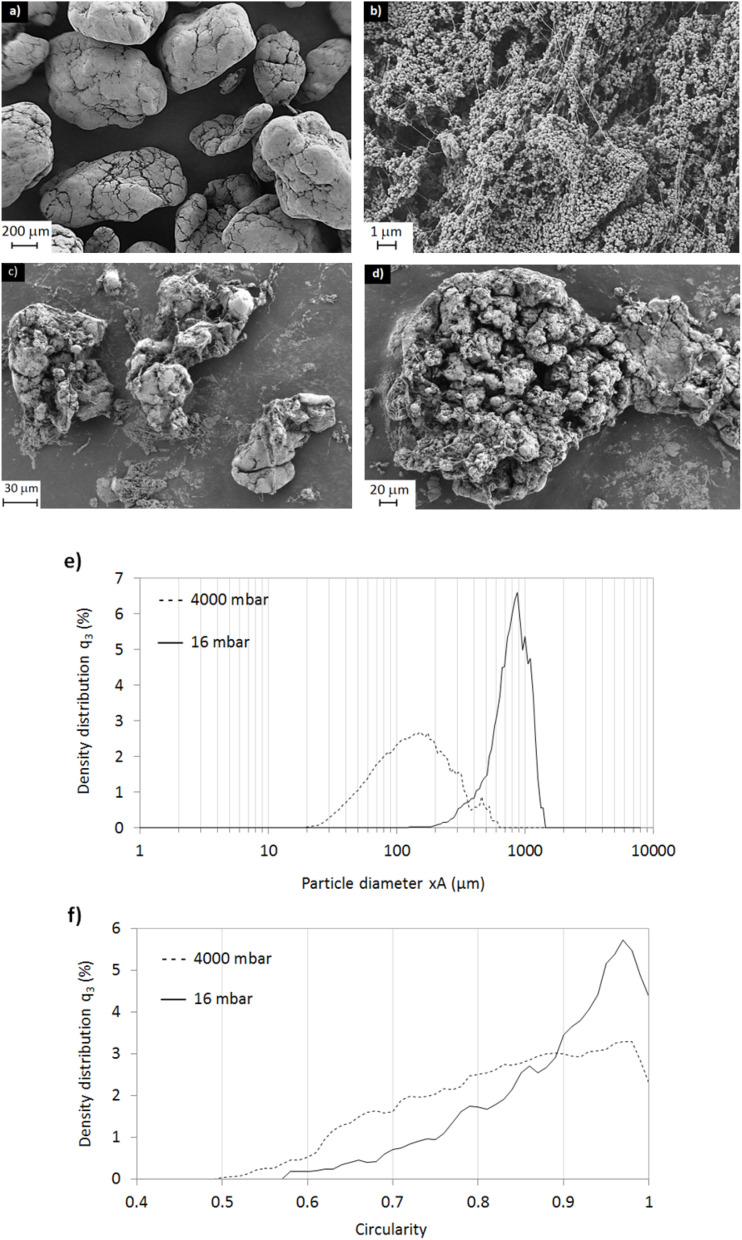
SEM images of as-received PTFE powder: (a) quasi-spherical granules (scale bar = 200 μm), (b) morphological make up of the granules (scale bar = 1 μm), (c) granules after 4000 mbar air-jet impact (scale bar = 30 μm), (d) a close-up of an individual air-impacted particle (scale bar = 20 μm), (e) particle size distributions of particles subjected to low (16 mbar) and high (4000 mbar) air pressures, (f) the effect of air pressure on particle shape (circularity parameter).

The as-received particles were imaged using SEM and shown to be quasi-spherical, with diameters in the approximate range 300–1000 μm (Fig. 6(a)). At higher magnifications, the morphology of these particles appeared as ∼100–150 nm spherical particles held in place by fibrous strands of polymer (Fig. 6(b)).

Dynamic image analysis was used to measure the particle size of the as-received particles, as shown in Fig. 6(e), with a volume-based mean *D*[4,3] measured as 724 μm. The particles were found to be extremely delicate and sensitive to handling. To further investigate this effect, the compressed air pressure of the dry-jet feed system was increased from 16 to 4000 mbar. Upon exposure to high pressures and shearing forces, the particles fragmented and became irregularly shaped with a shredded appearance (Fig. 6(c)). It's well known that above 19 °C, the cohesive forces between PTFE molecules weaken, making them more prone to being pulled apart and elongated by shear forces. Harris *et al.*^32^ propose that shear stress caused by sliding leads to chain scission of carbon bonds within the PTFE backbone. Wang *et al.*^33^ showed that PTFE particles were deformed into fine fibrils under shear or extensional flow during the extrusion process, due to the low interfacial shear strength of the particles. Fig. 6(d) above illustrates how the smoothness of the particles was lost as a result of high air pressure. As shown in Fig. 6(e), the large particles were broken down to particles sized 20–600 μm (*D*[4,3] = 147 μm).

Fig. 6(f) shows the effect of air pressure on particle circularity. For an average air pressure of 16 mbar, a high proportion of particles were in the range 0.9 to 1, *i.e.* with high circularity. By contrast, particles subjected to 4000 mbar had a lower proportion of particles in the range 0.9 to 1. They also had a higher proportion of particles in the range 0.5 to 0.9, *i.e.* lower circularity, relative to the 16 mbar sample. Those particles subjected to high air pressure were torn apart and characterised by rough morphologies and surface irregularities.

### 3.3 Fourier-transform infrared spectroscopy (FTIR)

**Fig. 7:**
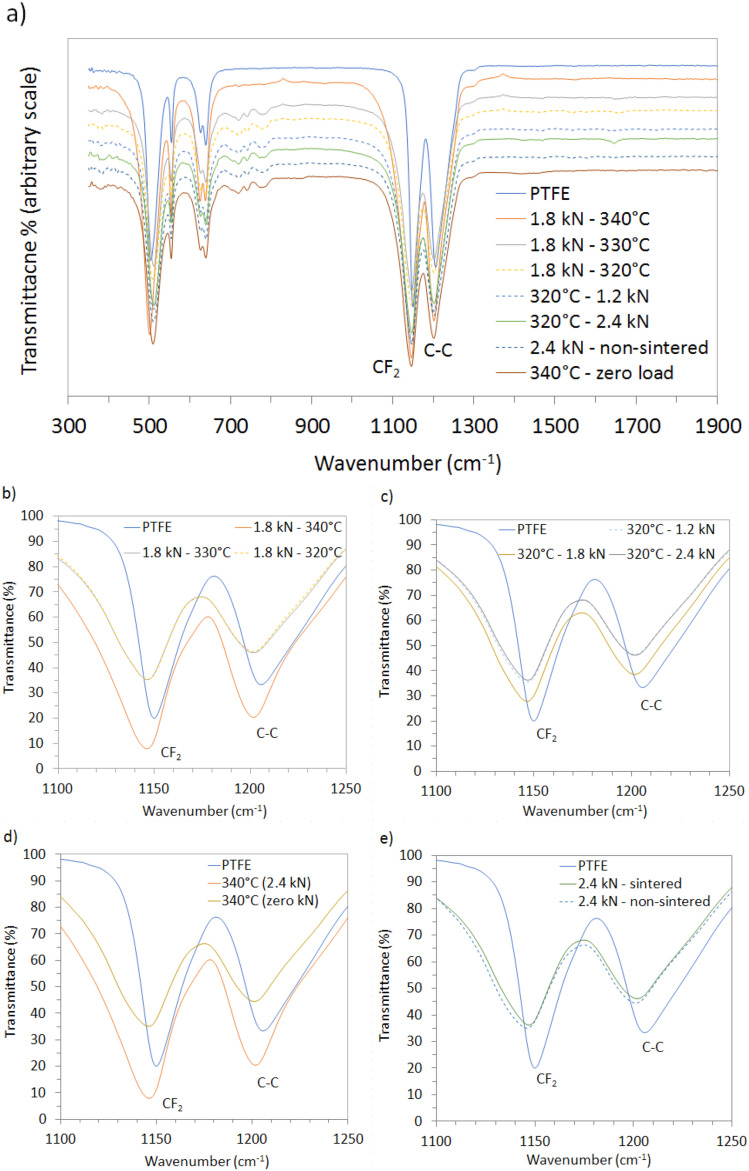
FTIR spectra of: (a) all samples processed at different temperatures and loads. (b) 2.4, 1.8, 1.2 kN, sintered at 320 °C, (c) 320, 330, 340 °C pressed at 1.8 kN, (d) 340 °C, with and without pressing and (e) 2.4 kN, with and without sintering.

FTIR spectra are shown in Fig. 7(a)–(e). The key absorption peaks are found at 1200 cm^−1^ and 1150 cm^−1^, corresponding to the CF_2_ stretching and C–C stretching modes, respectively.

CF_2_ stretching (≈1200 cm^−1^): this peak is attributed to the vibrational modes of the fluorine atoms bonded to the carbon atoms. Samples sintered at higher temperatures and/or compressed at higher loads exhibit an increase in CF_2_ peak intensity, indicating improved chain alignment and crystallinity. C–C stretching (≈1150 cm^−1^): this peak represents the carbon–carbon backbone of the polymer. The changes in intensity at this wavenumber similarly indicate modifications in chain packing and molecular organization and aligns with the findings of Tsai *et al.*^34^ The stability of peak positions observed suggests that the fundamental molecular structure of PTFE remains intact despite processing variations, corroborating the results of Piwowarczyk *et al.*^35^

Fig. 7(b) shows spectra for 1.8 kN and 320, 330 and 340 °C, respectively, revealing that the intensity of the CF_2_ and C–C peaks increases with higher temperatures, indicating a higher degree of crystallinity and chain ordering, in accordance with the X-ray diffraction observation discussed earlier.

The spectra of samples sintered at 320 °C and subjected to different loads (1.2, 1.8, 2.4 kN) are presented in Fig. 7(c). A slight intensification of the CF_2_ and C–C bands is observed with increasing load. This trend is consistent with marginal chain alignment and packing improvements, but, given the XRD uncertainty, should not be over-interpreted as a significant crystallinity gain.

Fig. 7(d) compares samples processed at a load of 1.8 kN with and without sintering at 340 °C. The non-sintered sample shows significantly lower peak intensities for both CF_2_ and C–C bonds, indicating less chain ordering and highlights how sintering facilitates the development of ordered crystalline structures.

Fig. 7(e) shows spectra of samples pressed at 2.4 kN, comparing a sintered sample (320 °C) with a non-sintered one. The sintered sample exhibits higher transmittance at the CF_2_ and C–C peaks. Although peak broadening is not pronounced, the shift to higher wavenumbers and the enhanced intensity for the sintered sample suggests greater ordering, whereas the non-sintered sample shows marginal shifts associated with a more amorphous structure. Notably, the spectrum of the sample processed at 2.4 kN exhibits a minor shift toward higher wavenumbers compared to the unsintered or lower-load samples, suggesting a change in the molecular environment, enhanced crystallinity, and alignment of polymer chains under higher loads.

### 3.4 Effect of temperature and load on porosity

**Fig. 8:**
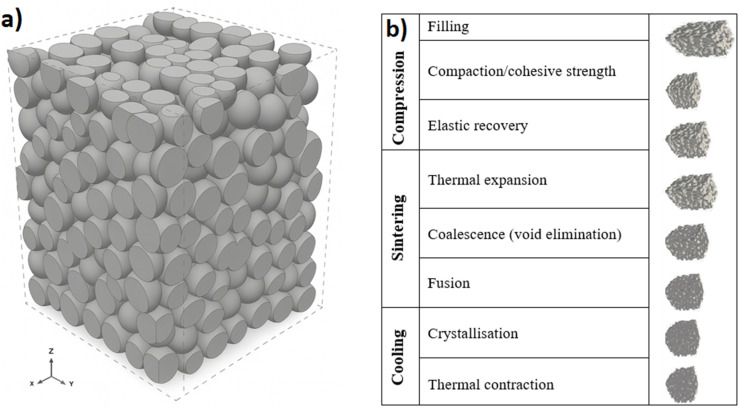
(a) Schematic of the formed 3D porous structure, and (b) the various stages of compression molding and sintering such a structure.

During pressing of PTFE powder followed by sintering, the particles will fuse together and form a film, as conceptualised in Fig. 8, below.

Fig. 8(a) depicts the pressed sample, prior to sintering. It assumes nearly monodisperse granules (∼500 μm diameter) initially arranged in a random close-packed bed (solid fraction ≈ 0.64, porosity ≈ 0.36). Under a load of 1.8 kN, the spherical particles progressively flatten at their contact points, increasing coordination number and driving the coalescence/void-elimination steps depicted in the right-hand column of Fig. 8(b). After the sieved powder is compacted, it recovers elastically upon removal from the mold, then undergoes thermal expansion during sintering. The consolidation, or coalescence, of particles during sintering produces a homogenous structure. The preform is then heated above the crystalline melting point of the powder, causing fusion, followed by a cooling cycle, crystallisation and thermal contraction.

**Fig. 9:**
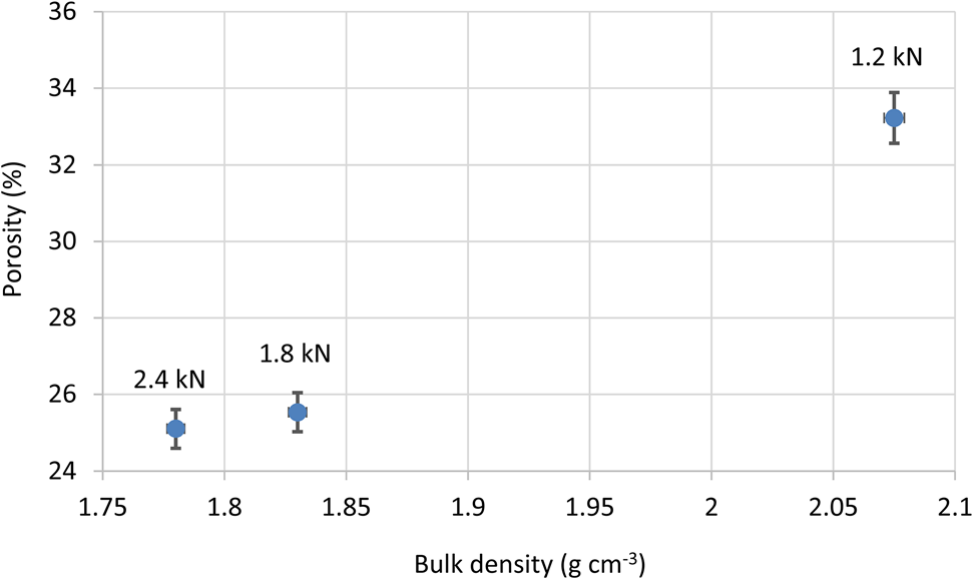
Porosity *versus* bulk density for samples compressed at 1.8 kN and sintered at 320 °C, 330 °C and 340 °C. Symbols are mean values of two replicate discs; error bars denote ±0.3% absolute porosity (1 SD).

The influence of temperature on porosity was investigated for a fixed compression load of 1.8 kN, as shown in Fig. 9. For each sintering temperature, two replicate discs (1.0 mm and 0.6 mm thickness) were tested; their porosity values (320 °C: 25.3% and 25.5%; 330 °C: 22.1% and 22.7%; 340 °C: 23.8% and 24.0%) differ by ≤0.4%, which is within the ±0.3% measurement uncertainty. This trend suggests that increasing the temperature enhances densification, likely due to improved chain mobility and particle coalescence reducing void spaces within the material.

The porosity increase at 340 °C is attributed to the onset of the melt phase at the applied pressure (as shown in Fig. 5), followed by void formation resulting from trapped gases or the coalescence of microvoids as the polymer transitions into a viscous or semi-fluid state.

The observed decrease in porosity with increasing temperature, from 320 to 330 °C, is consistent with better chain mobility and particle packing density. At 330 °C, the polymer chains achieve sufficient mobility for effective particle coalescence without significant degradation. However, at 340 °C, the onset of minor thermal decomposition or outgassing may reduce chain integrity and hinder densification. The relationship between compression load and porosity observed in our study is consistent with the findings of Van Rooyen *et al.*,^36^ who demonstrated that higher loads reduce void spaces, enhancing the density of PTFE. Yuan *et al.*,^31^ investigated the effect of sintering temperature on the crystallization behaviour of PTFE in SiO_2_-filled PTFE (PTFE/SiO_2_) composites sintered by hot pressing at temperatures of 350–390 °C. They observed dense surfaces at 350 and 370 °C, with porosity occurring on the surface as the temperature was raised to 390 °C. Meanwhile, the rod-like PTFE crystals became shorter and wider, along with indistinct grain boundaries and increased porosity. It is believed that the emergence of such crystals is favoured by high sintering temperature and slow cooling rate, in accordance with the result of Wang *et al.*^37^ Interestingly, our study highlights that even modest increases in load can lead to noticeable improvements in chain ordering. The role of load in enhancing chain alignment is also supported by the work of Frick *et al.*,^38^ who observed improved mechanical properties in PTFE for higher compression loads.

**Fig. 10:**
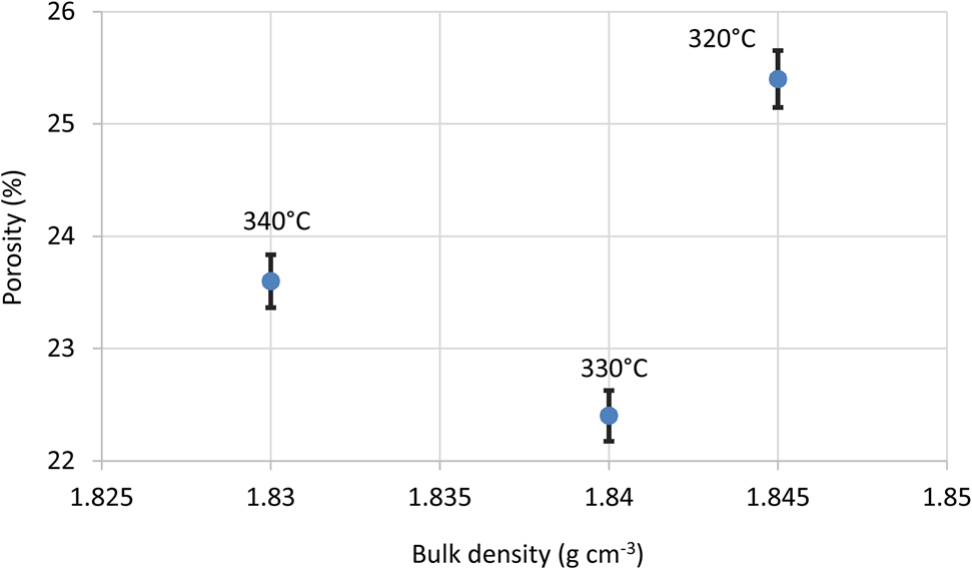
Porosity *versus* bulk density for PTFE films sintered at 320 °C and consolidated under compression loads of 1.2, 1.8, and 2.4 kN. Symbols represent the mean of two replicate discs (1.0 mm and 0.6 mm); error bars denote ±0.3% absolute porosity (1 SD).

The effect of varying loads on porosity at a constant temperature of 320 °C is shown in Fig. 10. 2.4 kN samples have values of 24.6% and 25.6% porosity, whereas 1.2 kN samples show values of 33.0% and 33.4%.

Fig. 9 and 10 demonstrate that both higher sintering temperatures and higher compression loads contribute to reduced porosity. The decrease, with increasing temperature, can be attributed to better particle coalescence and densification. Similarly, higher compression loads result in fewer voids. Notably, the mean porosity at 1.8 kN (25.5 ± 0.3%) differs from that at 2.4 kN (25.1 ± 0.3%) by only 0.4%, which lies within the ±0.3% experimental uncertainty. Therefore, any additional densification beyond 1.8 kN is statistically insignificant (*p* > 0.05).

### 3.5 Film morphology

**Fig. 11:**
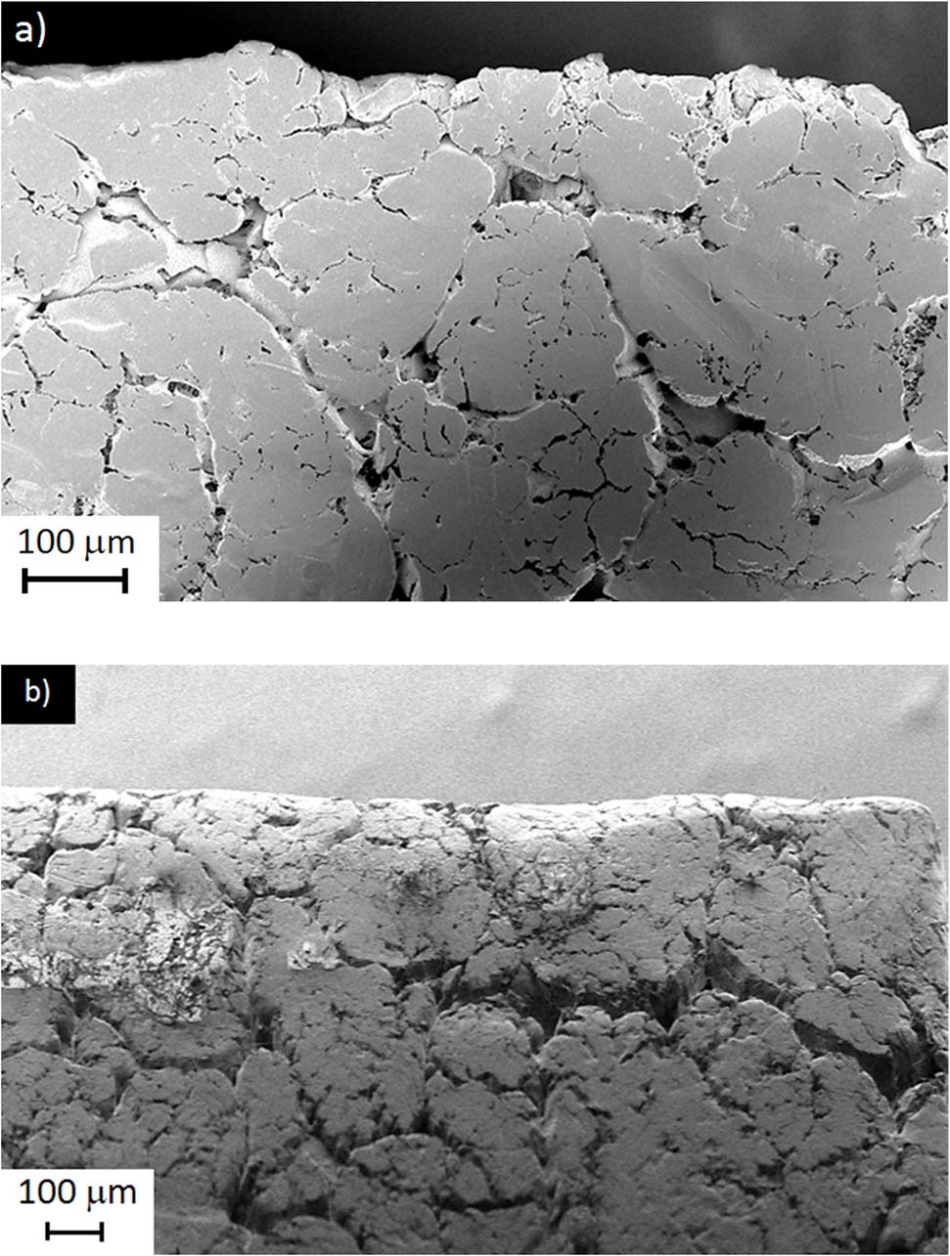
SEM images of (a) a polished cross-section and (b) the as-molded surface of films pressed at 1.8 kN and sintered at 340 °C.

Fig. 11(a) reveals a well-developed and continuous network of inter-particle necks, confirming that a high level of powder coalescence has been achieved. The particle size and morphology is reminiscent of the as-received particles seen in Fig. 6(a). Despite the delicacy of the powder during handling, there is no evidence of the particles being adversely affected by the compressive forces and/or sintering process. This is in part due to the low compression modulus and high degree of compressibility of PTFE particles. Tight packing and containment of the particles within the mold also serves to preserve their fundamental nature. This is in sharp contrast to the effects of shearing forces on the particles as observed in Fig. 6(c). However, the low interfacial shear strength of PTFE particles is considered an advantageous property for processes like PTFE paste extrusion, where the formation of a strong, fibrillar network acts as a reinforcing structure and provides the extrudate with strength and mechanical stability.

Angular interstices (dark crevices) are observed in Fig. 11, consistent with the measured ∼24% residual porosity. The understanding is that (i) sintering has shrunk the original void channels but not eliminated them, and (ii) the final pore location and shape has mirrored the initial inter-particle contacts. This would indicate that coalescence has proceeded mainly by local neck growth rather than long-range viscous flow. These observations corroborate the XRD/FTIR evidence of enhanced chain mobility at 340 °C, yet demonstrate that closed porosity is retained even under the highest temperature-load combination explored.

The as-molded surface morphology Fig. 11(b), contains larger cracks and voids relative to the cross-section, indicating that densification has proceeded from the bulk toward the free surface. The surface film exhibits a rough, micro-cracked skin, suggesting that trapped gases escaped through the top layer during cooling.

## 4 Conclusions

Compression-molding temperature is the primary driver of structural ordering in PTFE: raising the dwell from 320 °C to 340 °C (1.8 kN) boosts XRD-derived crystallinity by ∼8% and enlarges crystallite size by roughly 50%, reflecting improved chain mobility and crystal growth. FTIR and SAXS corroborate this progressive ordering while confirming retention of the phase-IV hexagonal lattice; the slight contraction of lattice parameters with cooling indicates tighter chain packing rather than a true phase change. Pressure, by contrast, chiefly controls densification. Raising the load from 1.2 kN to 2.4 kN at 320 °C reduces porosity by roughly a quarter yet produces crystallinity changes that are within experimental error, indicating that void volume is largely removed once moderate compression (∼1.8 kN) is reached. SEM cross-sections support these trends, revealing continuous sintering necks whose morphology mirrors the predicted particulate packing. Together, these findings define an efficient processing window, *i.e.* 330–340 °C under ∼1.8 kN, which maximises crystallinity and density without risking thermal degradation or demanding excessive press capacity, thus providing a practical guide for manufacturing high-performance PTFE components. Future work will link these microstructural metrics to mechanical strength and permeability to complete the PTFE process–property map.

## Conflicts of interest

There are no conflicts to declare.

## Data Availability

All data of this work have been included in the manuscript.
